# Rhino-orbito-cerebral mucormycosis caused by *Rhizopus microsporus var. microsporus* in a diabetic patient with COVID-19^⋆^

**DOI:** 10.1016/j.abd.2022.02.001

**Published:** 2022-06-09

**Authors:** Sandy Daniele Munhoz, Rute Facchini Lellis, Ana Paula Carvalho Reis, Gilda Maria Barbaro Del Negro, Maria Glória Teixeira Sousa, John Verrinder Veasey

**Affiliations:** a Dermatology Clinic, Hospital da Santa Casa de São Paulo, São Paulo, SP, Brazil; b Pathology Laboratory, Hospital da Santa Casa de São Paulo, São Paulo, SP, Brazil; c Medical Mycology Laboratory ‒ LIM-53, Division of Clinical Dermatology, Faculty of Medicine, Hospital das Clínicas and Instituto de Medicina Tropical de São Paulo, Universidade de São Paulo, São Paulo, SP, Brazil; d Medical Sciences College, Santa Casa de São Paulo, São Paulo, SP, Brazil

**Keywords:** Amphotericin B, Coronavirus, COVID-19, Diabetes *mellitus*, Diagnosis, Mucormycosis

## Abstract

COVID-19 disease caused by the SARS-CoV-2 coronavirus causes a wide range of clinical manifestations, ranging from mild to severe, with the main ones affecting the respiratory tract, such as pneumonia. In patients with greater severity, the high frequency of bacterial and fungal coinfection stands out, a situation related both to the patient's pre-existing comorbidities and due to the hospitalization itself. Cases of mucormycosis associated with COVID-19 were highlighted in the lay and scientific media, with the increase in mycosis cases being directly and indirectly attributed to the viral infection. This report describes a case of rhino-orbito-cerebral mucormycosis in a diabetic patient hospitalized for COVID-19, whose diagnosis was confirmed by identifying the agent *Rhizopus microsporus var. microsporus* through culture for fungi and PCR examination.

A 67-year-old male patient with diabetes mellitus (DM) reported eye pain and a decrease in visual acuity on the right eye for ten days, which started during a recent hospital stay in another hospital for COVID-19. His medical file reported treatment with corticosteroids, unspecified antibiotic therapy, and respiratory physical therapy. He was discharged from this hospital and immediately sought the service with edema and intense erythema on the right conjunctiva, mild erythema on the upper eyelid (Fig. 1), and ocular plegia on dynamic examination. Complementary tests showed DM decompensation, and a computed tomography showed inflammatory sinus disease. With the diagnostic hypothesis of rhino-orbito-cerebral mucormycosis, a surgical approach was performed, and the material was sent for histological examination, which showed coenocytic hyaline hyphae with a 90 ° angle (Fig. 2), and for fungal culture, which showed growth of *Rhizopus sp.* (Fig. 3). The colony was sent for identification of the isolate by PCR, and the regions of the internal transcribed spacer (ITS) were amplified using the ITS4 and ITS5 regions of the 5.8S gene of the fungus ribosomal DNA (rDNA), which identified the agent *Rhizopus microsporus var. microsporus*. After treatment with liposomal Amphotericin B and compensation of the underlying disease, the patient developed a worsening clinical picture, requiring a second surgical approach with ocular enucleation and extensive surgical debridement. The patient had a good evolution, with progressive improvement, and was discharged after 66 days of hospitalization under outpatient follow-up for aesthetic adjustment.

**Figure 1:**
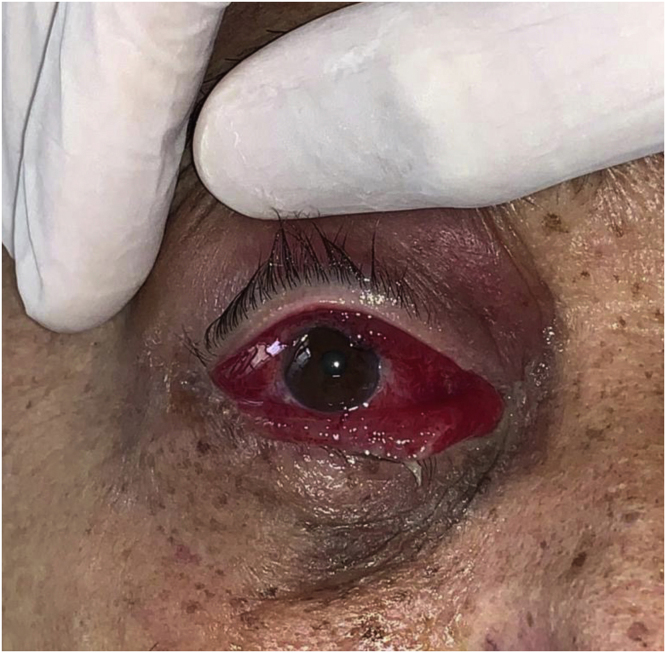
Erythema and severe swelling of the conjunctiva associated with upper eyelid erythema. Rhino-orbito-cerebral mucormycosis.

**Figure 2:**
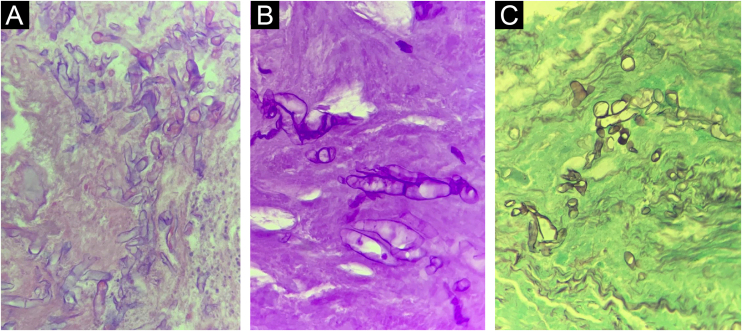
Histopathological section of a surgical specimen from the patient with rhino-orbito-cerebral mucormycosis showing coenocytic hyphae at ×400 magnification. (A), Hematoxylin & eosin. (B), PAS staining. (C), Grocott Gomori staining.

**Figure 3:**
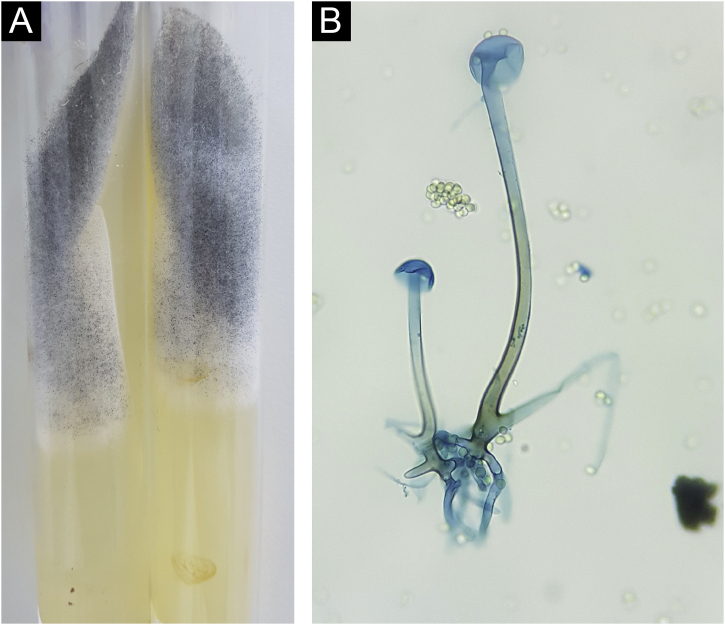
*Rhizopus microsporus*. (A), Colony consisting of whitish “cotton-wool” mycelium and blackish granular aerial mycelium. (B), Microculture showing coenocytic hypha with rhizoids, sporangiophore and empty sporangia. The presence of sporangiospores is observed around the structure (Cotton blue, ×400).

Mucormycosis is a rare, opportunistic, globally distributed fungal disease whose physiopathology is centered on angioinvasion that leads to tissue necrosis. Among the most often described etiological agents are the coenocytic fungi of the *Rhizopus* and *Mucor* genera.^1^, ^2^ Because it is an ubiquitous and saprophytic fungus, the infection usually occurs in patients with a disease that favors immunosuppression, with DM being the most common one.^2^, ^3^ The most frequent clinical form is the rhino-orbito-cerebral type,^1^, ^4^ which has a poor prognosis and shows better rates when amphotericin B is associated with extensive surgical excision of the infected tissue.^1^, ^5^

In a study conducted before the COVID-19 pandemic, 71% of the world's cases of mucormycosis were reported in India.^6^ In a recent study carried out in the same country, 1.8% of COVID-19 patients developed mucormycosis, of which 76.6% of these co-infected patients were diabetics.^7^ These rates are not surprising, as India ranks second in the world in absolute numbers of people with DM, which is the main risk factor for mucormycosis.^3^

SARS-CoV-2 also promotes changes in the host, especially in the severe forms of infection, favoring the development of mucormycosis agents. Among them are the direct damage to pancreatic cells, causing acute DM and ketoacidosis, the use of corticosteroids, which alter glycemic homeostasis and indirectly favor the progression of mycosis, the alteration of iron metabolism leading to an internal environment with high rates of ferritin, a key factor for the development of the fungus, and finally, endothelitis caused directly by the virus, facilitating fungal angioinvasion.^8^

The case described herein maintains the classic line of mucormycosis presentation, the rhino-orbito-cerebral type, in a patient with a history of DM, who shares another severity factor, COVID-19 infection. Prompt and correct treatment with antifungals and surgical approaches resulted in a disfiguring outcome, but with the preservation of the patient's life in this serious and fatal disease.

## Financial support

None declared.

## Authors' contributions

Sandy Daniele Munhoz: Design and planning of the study; data collection; analysis and interpretation of data; drafting and editing of the manuscript; obtaining, analyzing, and interpreting data; critical review of important intellectual content; intellectual participation in the propaedeutic and/or therapeutic conduct of the studied cases; critical review of the literature.

Rute Facchini Lellis: Analysis and interpretation of data.

Ana Paula Carvalho Reis: Analysis and interpretation of data.

Gilda Maria Barbaro Del Negro: Obtaining, analyzing and interpreting data.

Maria Glória Teixeira Sousa: Analysis and interpretation of data.

John Verrinder Veasey: Design and planning of the study; data collection; analysis and interpretation of data; drafting and editing of the manuscript; critical review of important intellectual content; effective participation in research orientation; intellectual participation in the propaedeutic and/or therapeutic conduct of the studied cases; critical review of the literature; approval of the final version of the manuscript.

## Conflicts of interest

None declared.
